# Single Port Robotic Extraperitoneal Distal Ureterectomy and Ureteral Reimplantation via Lower Anterior Approach

**DOI:** 10.1590/S1677-5538.IBJU.2026.0151

**Published:** 2026-03-15

**Authors:** Arianna Biasatti, Savio D. Pandolfo, Rocco Damiano, Marco De Sio, Ciro Imbimbo, Riccardo Autorino

**Affiliations:** 1 University of Trieste Department of Medicine Urological Clinic Trieste Italy Department of Medicine, Urological Clinic, Surgery and Health Sciences, University of Trieste, Trieste, Italy; 2 University of L’Aquila Department of Life Health and Environmental Sciences L’Aquila Italy Department of Life, Health and Environmental Sciences, University of L’Aquila, L’Aquila, Italy; 3 University of Naples "Federico II" Department of Neurosciences Sciences of Reproduction and Odontostomatology Naples Italy Department of Neurosciences, Sciences of Reproduction and Odontostomatology, University of Naples "Federico II", Naples, Italy; 4 Magna Graecia University of Catanzaro Department of Urology Catanzaro Italy Department of Urology, Magna Graecia University of Catanzaro, Catanzaro, Italy; 5 University of Campania "Luigi Vanvitelli" Department of Woman Child and General and Specialized Surgery Naples Italy Department of Woman, Child and General and Specialized Surgery, Urology Unit, University of Campania "Luigi Vanvitelli", Naples, Italy; 6 Glickman Urological Institute Cleveland Clinic Section of Robotic Surgery Cleveland OH United States Section of Robotic Surgery, Glickman Urological Institute, Cleveland Clinic, Cleveland, OH, United States

## Abstract

**Purpose:**

Single Port (SP) Robotic Extraperitoneal approach enhances maneuverability in the narrow extraperitoneal space while preserving peritoneal integrity (1, 2). First reports show reduced postoperative pain, fewer gastrointestinal complications and shorter hospital stay compared to multiport or open techniques (3-5). Recent experiences with robotic ureteral reconstruction have further supported the feasibility of advanced reconstructive procedures in selected patients (6-8) but literature on SP platform remains limited (9, 10), therefore we present this innovative technique.

**Materials and Methods:**

We report the case of a 44-year-old female (BMI 29.1 kg/m^2^), with medical history of fibromyalgia, who presented with right-sided back pain and nausea. CT-urogram showed severe right hydroureteronephrosis with a distal ureteral stricture and diffuse ureteral thickening, without a clear obstructing lesion. Diagnostic ureteroscopy revealed luminal narrowing due to wall thickening, without papillary masses. Biopsies and urine cytology were negative for urothelial carcinoma. A double-J stent was placed, and SP robotic distal ureterectomy with ureteral reimplantation was scheduled.

**Results:**

Low Anterior Access (LAA) was obtained through a 4-cm incision at the McBurney point. The retroperitoneal space was bluntly developed to identify the psoas muscle as anatomical landmark; the ureter was dissected caudally and resected at the level of the stricture. The bladder was then reached for cystotomy and ureteral reimplantation. Operative time was 160 minutes. No complications occurred and the patient was discharged on postoperative day 1. Final pathology revealed endometriosis. The ureteral stent was removed after 4 weeks; at 3-month follow-up the patient was asymptomatic, renal function was normal, and CT scan showed no right hydroureteronephrosis.

**Conclusion:**

Robotic SP extraperitoneal approach appears safe, feasible, and promising for ureteral reconstruction, combining retroperitoneal access with SP system dexterity and potentially reducing postoperative morbidity and hospital stay.

**Video 1:** Available at: VIDEO

## Data Availability

Data Not Provided

## References

[1] Lambertini L, Avesani G, Haberal HB, Torres Anguiano JR, Calvo RS, Morgantini L (2025). Single-port Robot-assisted Ureteral Reimplantation via Low Anterior Access: Step-by-step Procedure and Preliminary Comparative Results. Eur Urol Open Sci.

[2] Orsini A, Lasorsa F, Bignante G, Yoon J, Dymanus KA, Cherullo EE (2024). Single Port Robotic Nephrectomy via lower anterior retroperitoneal approach: feasible, safe and effective option in surgically complex patients. Int Braz J Urol.

[3] Ditonno F, Franco A, Licari LC, Bologna E, Manfredi C, Katz DO (2024). Implementation of single-port robotic urologic surgery: experience at a large academic center. J Robot Surg.

[4] Pulford C, Keating K, Rohloff M, Peifer D, Eames R, Maatman T (2021). How we do it: robotic-assisted distal ureterectomy with ureteral reimplantation. Int Braz J Urol.

[5] Ditonno F, Franco A, Manfredi C, Chow AK, Vourganti S, Cherullo EE (2023). Single Port Robotic Pyeloplasty: early single-center experience. Int Braz J Urol.

[6] Wang X, Meng C, Li D, Ying Y, Ma Y, Fan S (2024). Minimally invasive ureteroplasty with lingual mucosal graft for complex ureteral stricture: analysis of surgical and patient-reported outcomes. Int Braz J Urol.

[7] Amer-Mestre M, Tubau V, Guldris-García R, Rossello JB, Ayala EP (2024). Laparoscopic onlay-flap ureteroplasty using cecal appendix. Int Braz J Urol.

[8] Santa Cruz JAC, Danilovic A, Vicentini FC, Brito AH, Batagello CA, Marchini GS (2024). Ureteral access sheath. Does it improve the results of flexible ureteroscopy? A narrative review. Int Braz J Urol.

[9] Heo JE, Kang SK, Lee J, Koh D, Kim MS, Lee YS (2023). Outcomes of single-port robotic ureteral reconstruction using the da Vinci SP® system. Investig Clin Urol.

[10] Santarelli V, Valenzi FM, Haberal HB, Morgantini LA, Biasatti A, Aljoulani M (2025). Current status of single port robotic-assisted reconstructive urology: a systematic review, meta-analysis and structured summary of the available literature. J Robot Surg.

